# Imaging Assessment of Prognostic Parameters in Cases of Isolated Congenital Diaphragmatic Hernia: Integrative Review

**DOI:** 10.1055/s-0041-1740296

**Published:** 2022-05-27

**Authors:** Juliana da-Costa-Santos, João Renato Bennini

**Affiliations:** 1 Department of Maternal-Fetal Medicine, Universidade Estadual de Campinas, Campinas, SP, Brazil

**Keywords:** hernias diaphragmatic congenital, ultrasonography prenatal, magnetic resonance imaging, patient selection, reference standards, hérnias diafragmáticas congênitas, ultrassonografia pré-natal, imagem por ressonância magnética, seleção de pacientes, padrões de referência

## Abstract

**Objective** Antenatal recognition of severe cases of congenital diaphragmatic hernia (CDH) by ultrasound (US) and magnetic resonance imaging (MRI) may aid decisions regarding the indication of fetal endoscopic tracheal occlusion.

**Methods** An integrative review was performed. Searches in MEDLINE and EMBASE used terms related to CDH, diagnosis, MRI, and US. The inclusion criteria were reviews and guidelines approaching US and MRI markers of severity of CDH published in English in the past 10 years.

**Results** The search retrieved 712 studies, out of which 17 publications were included. The US parameters were stomach and liver positions, lung-to-head ratio (LHR), observed/expected LHR (o/e LHR), and quantitative lung index. The MRI parameters were total fetal lung volume (TFLV), observed/expected TFLV, relative fetal or percent predicted lung volumes, liver intrathoracic ratio, and modified McGoon index. None of the parameters was reported to be superior to the others.

**Conclusion** The most mentioned parameters were o/e LHR, LHR, liver position, o/e TFLV, and TFLV.

## Resumo

**Objetivo** A identificação pré-natal de casos graves de hérnia diafragmática congênita (HDC) por ultrassonografia (US) e ressonância magnética (RM) pode ajudar a decidir sobre a indicação de oclusão traqueal endoscópica fetal.

**Métodos** Uma revisão integrativa foi realizada pesquisando nas bases MEDLINE e EMBASE com termos relativos a HDC, diagnóstico, RM, e US. Os critérios de inclusão foram revisões e diretrizes abordando marcadores ultrassonográficos e de ressonância para a gravidade de HDC publicados em inglês nos últimos 10 anos.

**Resultados** Foram obtidos 712 estudos, dos quais 17 foram incluídos. Os parâmetros de US foram posições do estômago e do fígado, relação pulmão-cabeça (LHR, na sigla em inglês), LHR observada/esperada (o/e LHR), e índice pulmonar quantitativo (QLI, na sigla em inglês). Os parâmetros de RM foram volume pulmonar fetal total (TFLV, na sigla em inglês), o/e TFLV, volume pulmonar fetal relativo e porcentagem predita, razão do fígado intratorácico (LiTR, na sigla em inglês) e índice de McGoon modificado. Nenhum dos parâmetros foi mencionado como superior aos demais.

**Conclusão** Os parâmetros mais citados foram o/e LHR, LHR, posição do fígado, o/e TFLV, e TFLV.

## Introduction

Congenital diaphragmatic hernia (CDH) is a rare disease, 1 with a reported frequency of 1 to 4 per 10,000 live births. 2 3 4 5 6

Congenital diaphragmatic hernia is an embryological defect derived from the incomplete fusion of the septum transversum, pleuroperitoneal folds, esophageal mesentery, and muscles from the body wall. 3 The defect may be located anywhere in the diaphragm, but it is usually located in the posterolateral region: Bochdalek hernia, corresponding to up to 95% of cases. 3 Also, left-sided hernias constitute the majority of cases (85%). 1

Congenital diaphragmatic hernia results in the displacement of abdominal organs, which mainly includes different combinations of herniated intestines, stomach, and/or liver. 3 4 Thus, a mass effect is created in the thoracic cavity, reducing lung growth and resulting in variable degrees of pulmonary hypoplasia and pulmonary hypertension, which are the main causes of postnatal severe morbidity and mortality. 4 6 In addition, considering the need for postnatal definitive surgical intervention for CDH and for neonatal intensive care, delivery should occur in a tertiary center. 4

Since the 1980s, it is possible to recognize cases of CDH with antenatal ultrasound. 7 The ability to identify CDH antenatally, and the frequency of neonatal complications of these infants led to the development of an intrauterine intervention: fetal endoscopic tracheal occlusion (FETO). This procedure cannot substitute the postnatal intervention, for it does not repair the diaphragmatic defect, but it enhances lung development by preventing lung fluid escape, thus increasing intrapulmonary pressure, cellular proliferation, and allowing maturation of the pulmonary vasculature. 2

Fetal endoscopic tracheal occlusion is a surgical intervention. Therefore, patient selection is of utmost importance. Currently, FETO is reserved for cases with poor prognosis, 5 but there is no consensus on which parameters should be used for prognostic assessment. Ultrasound (US) and magnetic resonance imaging (MRI) criteria have been used heterogeneously in the literature to classify cases of CDH, with numerous techniques and cutoff points. Therefore, the present study aimed to review the antenatal US and MRI criteria to assess the severity of the disease (CDH), their cutoff points, and postnatal prognosis according to the literature.

## Methods

An integrative review was performed according to Whittemore and Knafl. 8

A search was conducted in the MEDLINE and EMBASE databases, using the following strategies:

- MEDLINE: [ *Hernias* , *Diaphragmatic* , *Congenital* AND *Diagnosis* AND ( *Ultrasonography* , *Prenatal* ” OR *Magnetic Resonance Imaging* )]. Filter: past 10 years.
- EMBASE: *congenital diaphragm hernia* AND *diagnosis* AND ( *ecography* OR *nuclear magnetic resonance* ) AND [2011–2021]/py.

The inclusion criteria were reviews and protocols approaching US and MRI prognostic markers of severity of disease published in English in the past 10 years. Clinical studies of any design and reviews and/or protocols that did not address antenatal imaging prognostic tools were excluded. Studies published in other languages were excluded. Data was collected in an online form specifically developed for this review.

Study selection was performed independently by the authors (J. C. S. and J. R. B.) using Rayyan software (Rayyan Systems Inc., Cambridge, MA, USA). 9 Conflicting selections were resolved by consensus. The first step of study selection was screening by title and abstract. The publications included were further screened by full text assessment. For each parameter, the severity of disease was defined according to the reported outcome in each review, such as mortality and pulmonary hypertension.

## Results

**Fig. 1:**
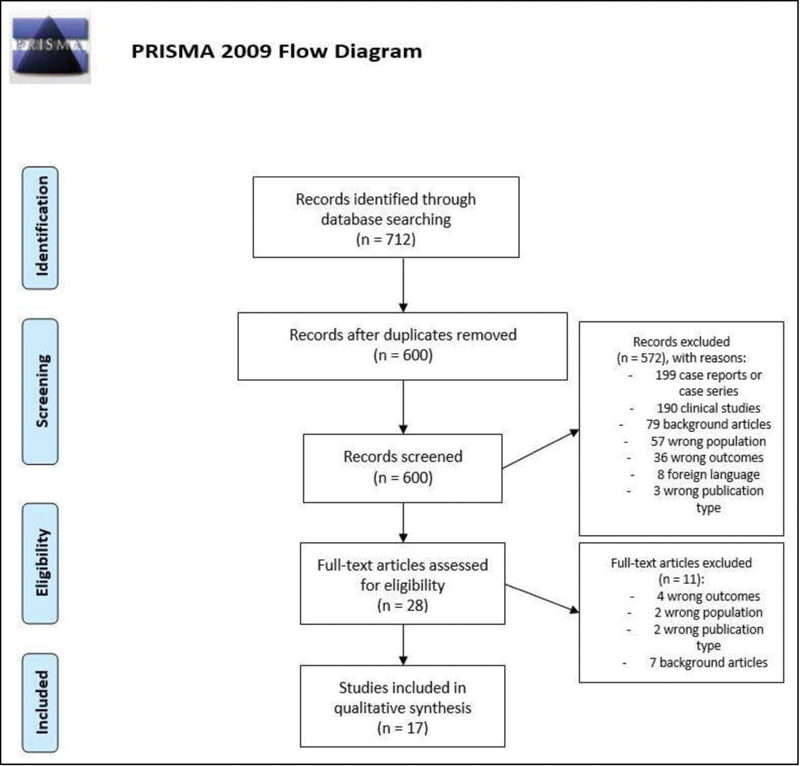
Preferred Reporting Items for Systematic Reviews and Meta-Analyses (PRISMA) flow diagram of study selection.

The search retrieved 712 studies. After the exclusion of duplicates ( *n* = 112), 600 studies were independently screened by J. C. S. and J. R. B. by title and abstract. The first step of study selection resulted in inclusion of 22 studies. There were 49 conflicting selections, which were resolved by consensus, and resulted in the inclusion of 6 of these publications. The second stage of study selection was full text assessment of the 28 studies included. In this step, 16 studies were included, and there were 2 conflicting selections, which were resolved by consensus, and 1 article was included, totaling 17 studies for this review. The main reasons for exclusion are described in Figure 1 . The final selection included 17 studies, which analyzed prenatal US and MRI parameters for prediction of severity of the disease. They included 14 narrative reviews (of these, 1 was a narrative review and clinical protocol), 1 2 3 4 5 7 10 11 12 13 14 15 16 17 2 systematic reviews with metanalyses, 18 19 and 1 clinical protocol. 6 Regarding the imaging method, one of the studies approached MRI exclusively, 19 7 analyzed exclusively US, 1 2 3 4 6 13 14 and 9 studies reported MRI and US parameters. 5 7 10 11 12 15 16 17 18 Although the imaging modality varied throughout the selected studies, it is important to note that the severity of disease definitions and reference cutoff points for each parameter were similar; this is probably due to the use of the same source of information. There was a notable heterogeneity of gestational ages (GAs) at the time of the evaluation: 6 studies did not report GA at the imaging evaluation, 3 10 13 14 17 18 and the other 11 studies reported GA that ranged from 18 to 39 weeks. 1 2 4 5 6 7 11 12 15 16 19 There were five US and six MRI parameters described in the reviews. The US parameters were stomach position, liver position, lung-to-head ratio (LHR), observed/expected LHR (o/e LHR), and quantitative lung index (QLI). The MRI parameters were total fetal lung volume (TFLV), observed/expected TFLV (o/e TFLV), relative fetal lung volume (relative FLV), percent predicted lung volume (PPLV), liver intrathoracic ratio (LiTR), and modified McGoon index. The findings of this review are summarized in Tables 1 and 2 .

**Table 1:** Summary of ultrasound parameters for antenatal evaluation of severity of disease in CDH

US parameter	Number of reviews that mentioned the parameter	Lesion side	GA at evaluation (weeks)	Cutoff point for severe CDH	Definition of severity
Stomach position	4	Left	≥ 22	Stomach up	Survival: stomach up: 29–35%; stomach down: 90–100%
					Survival according to grade of herniation: grade 1 and grade 2: around 90%; grade 3: around 70%; grade 4: around 10%
Liver position	8	Left	Not mentioned	Liver up	Mortality liver up: 44–100% Liver down: 0–45.4%
					Need for ECMO Liver up: 42–80% Liver down: 0–25%
LHR	8	Left	< 25	LHR < 0.6 and liver up	Survival rates of liver up and LHR: < 0.6: 0 0.6–1.35: 61% > 1.4: 100%
		Not mentioned	20–26	< 1.0	Survival: < 0.6: 0 > 1.35: 100%
o/e LHR	16	Left	18–38	< 25%	Survival: o/e LHR > 55%: 85% (liver up)–100% (liver down)
					46–55%: 90% (liver up) to 100% (liver down)
					36–45%: 65% (liver up) to 75% (liver down)
					26–35%: 55% (liver up) to 65% (liver down)
					≤ 25%: 15% (liver up)–30% (liver down)
		Right	18–38	< 45%	Survival rate: < 45%: up to 17%
					> 45%: at least 60%
QLI	1	Not mentioned	Not mentioned	QLI < 0.6 (or centile 1)	Values correlate with lung hypoplasia

Abbreviations: CDH, congenital diaphragmatic hernia; ECMO, extracorporeal membrane oxygenation; LHR, lung-to-head ratio; o/e, observed/expected; QLI, quantitative lung index; US, ultrasound.

**Table 2:** Summary of magnetic resonance imaging parameters for antenatal evaluation of severity of disease in CDH

MRI parameter	Number of reviews that mentioned the parameter	Lesion side	GA at evaluation (weeks)	Cutoff point for severe CDH	Definition of severity
TFLV	5	Right and left	18–38	< 20cm ^3^	Survival: TFLV < 20 cm ^3^ : 35% TFLV > 40 cm ^3^ : 90%
					Need for ECMO: TFLV < 20 cm ^3^ : 86% TFLV > 40 cm ^3^ : 10%
o/e TFLV	10	Right and left	≥ 22	25%	Survival: o/e TFLV < 25%: 25% or less o/e TFLV 25–35%: 25–69% o/e TFLV > 35%: 75 - 89%
Relative FLV	1	Not mentioned	Not mentioned	40%	Values correlate with poor outcomes
PPLV	3	Not mentioned	Not mentioned	15%	Survival: PPLV < 15%: 40% PPLV > 40%: survival 100%
LiTR	2	Not mentioned	Not mentioned	Liver herniation of 14% or more:	Values correlate with mortality
Modified McGoon index	1	Not mentioned	Not mentioned	1.0	Values correlate with severe pulmonary hypertension at 3 weeks of life

Abbreviations: CDH, congenital diaphragmatic hernia; ECMO, extracorporeal membrane oxygenation; FLV, fetal lung volume; GA, gestational age; LiTR, liver intrathoracic ratio; MRI, magnetic resonance imaging; o/e, observed/expected; PPLV, percent predicted lung volume; TFLV, total fetal lung volume.

US parameters.

### Stomach Position

There were five reviews describing this criterion. 1 5 7 10 12 The parameter was mostly reported in the context of left sided hernias and evaluated at 22 weeks of gestational age (GA) or more. There were two different descriptions of severity: the first one considered two possibilities of stomach position (abdominal — “stomach down”— or thoracic — “stomach up”), and the second one considered grades of stomach herniation. Stomach up was associated with survival rates of 29 to 35%, whereas stomach down was associated with survival rates of 90 to 100%. Stomach herniation grades evaluate stomach position in relation to thoracic organs: grade 1 is characterized by stomach in the normal position; grade 2 is characterized by stomach anteriorly at the apex of the heart and in contact with the chest wall; grade 3 has the larger part of the stomach anteriorly, but it reaches the level of the atrioventricular valves; and grade 4 has most of the stomach in the posterior thorax, in a retrocardiac position. According to this classification, survival rates are around 90% for grades 1 and 2, around 70% for grade 3, and around 10% for grade 4.

### Liver Position

There were eight studies describing this parameter. 1 4 5 7 10 11 17 18 Liver position could be abdominal (“liver down”) or thoracic (“liver up”). None of the reviews mentioned the GA for this assessment. Liver up was associated with greater mortality rates (44–100%), contrasting with liver down (0–45.4%). Furthermore, liver herniation is associated with the need for extracorporeal membrane oxygenation (ECMO); in liver-down cases, the need for ECMO ranged from 0 to 25%, whereas liver-up cases needed ECMO in 42 to 80% of cases.

### Lung-to-head Ratio: LHR

The search retrieved eight reviews. 1 3 4 5 10 11 15 18 Lung-to-head ratio is obtained by dividing the area of the lung contralateral to the hernia at the level of the four chambers view by the head circumference. Lung area measurement can be performed by obtaining the greatest orthogonal diameters at the level of the mid-clavicle line or at the level of the atrioventricular valves, or tracing the contours of the lung. 1 2 10 16 17 The tracing method is the most reproducible and accurate method for this parameter. 1 2 6 12 16 17 Reviews approaching left sided CDH with LHR used this ratio before 25 weeks of gestation and found that, if the LHR is less than 0.6 and there is liver herniation, there are no survivors; if LHR is 0.6 to 1.35, survival rate is 61%, and, when LHR is > 1.4, survival rate is 100%. When the side of the lesion is not mentioned, the most common cutoff point was less than 1.0 for poor outcomes such as mortality, need for ECMO, and lung hypoplasia.

### Observed/Expected LHR: O/E LHR

Observed/expected LHR was mentioned in 16 studies. 1 2 3 4 5 6 7 10 11 12 13 14 15 16 17 18 Regardless of the side of the lesion, the o/e LHR was evaluated between 18 and 38 weeks. Observed/expected LHR is obtained by dividing the LHR of the examined fetus by the expected LHR for that GA. For left sided lesions, there are multiple ways of classifying the disease. The general concept is that there are subgroups of o/e LHR which correlate with mortality and, given the same subgroup of o/e LHR, liver herniation could enhance mortality. The most used cutoff point for severe disease was o/e LHR less than 25%. When o/e LHR is ≤ 25% and the liver is herniated (liver up), the estimated survival rate is 15%. For the same o/e LHR, if the liver is down, survival could reach 30%. If the o/e LHR is between 26 and 35% and the liver is up, survival rate is 55%; if the liver is down, survival rate is 65%. For o/e LHR between 36 and 45% and liver up, estimated survival rate is 65%. In the same subgroup, if the liver is down, survival is estimated to be 75%. When the o/e LHR is between 46 and 55%, survival rates are estimated to be 90% if the liver is up, and 100% if the liver is down. Finally, if the o/e LHR is greater than 55%, survival rates are estimated to be 85% if the liver is up and 100% if the liver is down. For right sided lesions, the reported cutoff point for severe disease is 45%. For o/e LHR greater than 45%, survival rate is at least 60%. For o/e LHR less than 45%, survival rate is up to 17%. Liver herniation, in this case, is not a prognostic factor because this type of hernia usually presents with liver up.

### Quantitative Lung Index: QLI

The search retrieved one study that presented QLI as a parameter for evaluation of severity of the disease. 5 The present study did not report GA at evaluation or side of the lesion. Quantitative lung index is obtained by the division of the contralateral lung area by a tenth of the head circumference. Values below the 1 ^st^ percentile, which corresponds to QLI 0.6, are predictive of lung hypoplasia.

## MRI Parameters

### Total Fetal Lung Volume: TFLV

Total fetal lung volume is the sum of both lung volumes obtained by MRI, which was mentioned as a severity of disease marker by 5 studies. 5 6 11 18 19 Total fetal lung volume was used to evaluate both left- and right-sided lesions. The GA at evaluation was variable, from 18 to 38 weeks. The cutoff point of TFLV less than 20 cm ^3^ was suggested. When the TFLV is greater than 40 cm ^3^ , survival is estimated to be 90%, and the need for ECMO is around 10% of cases. When the TFLV is below 20 cm ^3^ , survival is up to 35% and 86% of these infants need ECMO. The TFLV was evaluated in some studies, with different populations, at variable GAs, aiming to elaborate reference values. 11 19 This topic was previously addressed, 20 and it was observed that normal and abnormal lung volumes at a certain GA could overlap. Furthermore, the range of normality widens throughout gestation. For example, at 25 weeks of gestation, the normal range varies from 20 to 35 cm ^3^ , and, at 35 weeks of gestation, it varies from 58 to 95 cm ^3^ . Therefore, the TFLV is generally used to obtain the o/e TFLV.

### Observed/Expected TFLV: o/e TFLV

This criterion was reported in 10 studies. 5 7 10 11 12 15 16 17 18 19 This parameter is the MRI analog of o/e LHR. The suggested cutoff point is 25%: below this threshold, survival rate is 25% or less. When o/e TFLV is between 25 and 35%, survival is reported as 25 to 69%. Survival of 75 to 89% is obtained when o/e TFLV is greater than 35%. Evaluation of the o/e TFLV is feasible for right- and left-sided lesions at a GA of 22 weeks or more.

### Relative Fetal Lung Volume: Relative FLV

Relative FLV was cited in one study. 10 A relative FLV of 40% or less correlates with poor outcomes. Gestational age at the evaluation and side of the lesion were not mentioned.

### Percent Predicted Lung Volume: PPLV

Percent predicted lung volume was reported by 3 studies. 5 10 18 The suggested cutoff point for severe disease was less than 15%. Below this threshold, survival rates are estimated to be 40%, and above this threshold, the survival rate is 100%. There was no mention of GA at evaluation or side of the lesion.

### Liver Intrathoracic Ratio: LiTR

The search retrieved two studies that mentioned LiTR. 5 18 None of the studies mentioned either the GA for assessment or the side of the lesion. This parameter is a ratio between herniated liver volume and fetal thoracic volume. Liver intrathoracic ratio of 14% or greater correlates with neonatal mortality.

### Modified McGoon Index

The modified McGoon index is a mathematical relation involving the sum of the diameters of the right and left pulmonary arteries, which are then divided by the diameter of the aorta. This parameter was assessed by one study. 10 Values lower than 1.0 correlate with severe pulmonary hypertension at 3 weeks of life. Side of the lesion and GA at evaluation were not mentioned.

## Conclusion

Several parameters were evaluated for severity of CDH assessment throughout the past 10 years. Although none of the parameters was reported to be superior to the others, the most mentioned ones were o/e LHR, LHR, liver position, o/e TFLV, and TFLV. This information may be used as the background for other studies comparing the performance of different diagnostic techniques.
